# A Single-Arm Phase 2 Trial on Induction Chemotherapy Followed by Concurrent Chemoradiation in Nasopharyngeal Carcinoma Using a Reduced Cumulative Dose of Cisplatin

**DOI:** 10.3389/fonc.2022.842281

**Published:** 2022-04-27

**Authors:** Zhiyuan Xu, Li Yang, Wai-Tong Ng, Aya El Helali, Victor Ho-Fun Lee, Lingyu Ma, Qin Liu, Jishi Li, Lin Shen, Jijie Huang, Jiandong Zha, Cheng Zhou, Anne W. M. Lee, Longhua Chen

**Affiliations:** ^1^Department of Radiation Oncology, Nanfang Hospital, Southern Medical University, Guangzhou, China; ^2^Clinical Oncology Centre, The University of Hong Kong - Shenzhen Hospital, Shenzhen, China; ^3^Department of Clinical Oncology, The University of Hong Kong, Hong Kong, Hong Kong SAR, China

**Keywords:** nasopharyngeal carcinoma, induction chemotherapy, cisplatin, capecitabine, progression-free survival

## Abstract

**Background:**

We conducted this study to evaluate if a reduced cumulative dose of induction and concurrent cisplatin conferred similar favorable outcomes when compared to trial NPC-0501.

**Methods:**

Newly diagnosed nasopharyngeal carcinoma (NPC) with stage III-IVA were prospectively recruited from January 2015 to September 2019. Induction chemotherapy (IC) consisted of cisplatin 80mg/m^2^ on day 1 and capecitabine 1000mg/m^2^ twice daily from day 1 to 14 every 3 weeks for 3 cycles followed by concurrent chemoradiotherapy (CCRT) with 2 cycles of cisplatin 100mg/m^2^ given every 3 weeks. Tumor response was evaluated according to RECIST v1.1. Acute and late adverse events (AEs) were graded with CTCAE v4.0 and Late Radiation Morbidity Scoring of the RTOG, respectively.

**Results:**

135 patients were recruited. At 16 weeks after CCRT, all 130 patients who completed the entire course of radiotherapy (RT) had a complete response upon final assessment. With a median follow-up of 36.2 months, 22 treatment failures and 8 deaths were observed. The 3-year progression-free survival, overall survival, locoregional recurrence-free survival, and distant recurrence-free survival were 83.7%, 94.1%, 94.1%, and 85.9%, respectively. Our survival data outcomes were similar to those reported in the cisplatin and capecitabine (PX) induction arm of the 0501 trial. 103 patients (76.3%) reported acute grade 3-4 AEs. Two patients (1.5%) had late grade 3-4 complications, numerically fewer than those reported in the NPC-0501 trial.

**Conclusions:**

Induction PX and concurrent cisplatin with a reduced cumulative cisplatin dose yield survival outcomes comparable to those reported in the NPC-0501 trial with excellent tolerability. Therefore, a reduced cumulative dose of cisplatin is a promising treatment scheme for nasopharyngeal carcinoma.

## Introduction

Nasopharyngeal carcinoma (NPC) is an endemic malignancy with a specific geographical distribution. It will affect an estimated 133,354 patients worldwide in 2020, with the highest incidences occurring in South China, Southeast Asia, and North Africa (1, 2). More than 70% of NPC patients have locoregionally advanced disease at the time of presentation (3). Intensity-modulated radiation therapy (IMRT) with concurrent platinum-based chemotherapy constitutes the backbone of treatment for locoregionally advanced nasopharyngeal carcinoma (LA NPC). Although the locoregional control rate in NPC has been substantially improved, distant metastasis remains the predominant pattern of treatment failure (4).

The addition of chemotherapy as induction or adjuvant regimen to concurrent chemoradiotherapy (CCRT) has been extensively investigated. Since the first report of significant survival benefits by the Intergroup 0099 study (5), the addition of adjuvant cisplatin and 5-fluorouracil (PF) to CCRT has become a standard of care recommendation for patients with LA NPC (6). However, a significant concern regarding the concurrent-adjuvant approach is poor compliance (approximately 60%) to three cycles of adjuvant chemotherapy (7). Compared with adjuvant chemotherapy (AC), induction chemotherapy (IC) offers improved tolerability, early eradication of micrometastases, wider margin, and better radiation coverage during subsequent CCRT. A phase 3 randomized controlled trial in Hong Kong (NPC-0501) evaluated the therapeutic gain of changing the chemotherapy sequence from concurrent-adjuvant to induction-concurrent and replacing 5-fluorouracil with capecitabine for patients with LA NPC (7, 8). This trial revealed that changing the chemotherapy sequence from a concurrent-adjuvant to an induction-concurrent sequence could improve efficacy without adversely impacting toxicities. Furthermore, replacing 5- fluorouracil with capecitabine significantly lowered the risk of progression and death. Induction cisplatin plus capecitabine (PX) incurred fewer toxicities such as neutropenia and electrolyte disturbance than induction PF (7, 8). In addition, capecitabine has shown a promising survival benefit in maintenance therapy for metastatic nasopharyngeal carcinoma (9). However, the switch from 5-fluorouracil to oral capecitabine warrants further validation given its convenience, favorable toxicity profile, and favorable trends in efficacy.

Patients allocated to the induction-PX arm in the NPC-0501 trial received induction cisplatin 100 mg/m^2^ on day 1 plus capecitabine 1000 mg/m ^2^ twice daily on days 1 to 14 every 21 days for 3 cycles and concurrent cisplatin 100mg/m^2^ on day 1 every 21 days for 3 cycles. The proportion of patients that received 3 concurrent cycles was 33% in the induction-PX arm. Most induction platinum-based doublet chemotherapy regimens implemented a cisplatin dose of 75-80mg/m^2^ for 2 to 3 cycles (10–12). Furthermore, some evidence suggested that a cumulative cisplatin dose of 200 mg/m^2^ during CCRT may be adequate to achieve a survival benefit (13, 14). However, whether or not a reduced cumulative cisplatin dose in both induction PX and the CCRT phases provide comparable treatment outcomes to that reported in the NPC-0501 trial remains unclear. Therefore, we conducted this prospective, single-arm, phase 2 trial to investigate the efficacy and safety of reduced cumulative cisplatin in PX induction chemotherapy and CCRT in LA NPC.

## Methods

### Study Design and Patients

This study was a prospective, single-arm, phase 2 trial conducted in a single institute in China. Eligibility was defined as newly diagnosed, previously untreated, histologically confirmed non-keratinizing NPC, stage III-IVB disease as per the 7th edition of the American Joint Committee on Cancer–Union for International Cancer Control (AJCC-UICC TNM-7) for patients diagnosed before 2018 or stage III-IVA disease as per AJCC-UICC TNM-8 for patients diagnosed on or after 2018 (except T3N0). Re-staging was performed using AJCC-UICC TNM-8 for patients enrolled prior to 2018 by two independent oncologists before the final analyses of this study. Any discrepancy in staging was resolved by consensus. Other inclusion criteria were age 18 to 75 years, an Eastern Cooperative Oncology Group (ECOG) performance status (PS) ≤ 2, adequate hematologic, hepatic, and renal function. Key exclusion criteria were the following: treatment for palliative intent; a history of prior malignancy; a history of previous chemotherapy, radiotherapy, or surgery (except diagnostic procedures) to the primary tumor or nodes; pregnancy or lactation; or any severe comorbidity. The local institutional ethics committee approved the trial protocol (reference number 201627). The trial was conducted according to the Declaration of Helsinki and Good Clinical Practice guidelines. All patients provided written informed consent before enrollment. Patients could withdraw consent at any time after enrollment. This trial is registered on clinicaltrials.gov as NCT03427359, (https://clinicaltrials.gov/ct2/show/NCT03427359?term=NCT03427359&draw=2&rank=1).

Pre-treatment assessment included the following: complete history and physical examination; complete blood count, renal and liver function tests; Epstein-Barr virus- deoxyribonucleic acid (EBV-DNA) test; dental, audiometric, and nutritional assessment; fiberoptic nasopharyngoscopy; magnetic resonance imaging (MRI) or contrast-enhanced computed tomography (CT) of the head and neck region (if MRI was contraindicated) for primary tumor staging; contrast-enhanced CT of the chest and abdomen, together with skeletal scintigraphy for distant metastasis staging. 18F-fluorodeoxyglucose-positron-emission tomography with integrated computed tomography (PET-CT) scan was recommended though not mandatory.

### Treatment and Assessment

Patients received induction PX with cisplatin at a dose of 80 mg/m^2^ as an intravenous infusion on day 1 plus oral capecitabine at a dose of 1000 mg/m^2^ twice daily from day 1 to 14 every 21 days for 3 cycles. In the CCRT phase, cisplatin was delivered concurrently with radiotherapy (RT) and administered intravenously at a dose of 100 mg/m^2^ on days 1 and 22 for 2 cycles. Details of the chemotherapy dose modifications are available in the **Supplementary Appendix**.

Treatment with intensity-modulated radiotherapy (IMRT) or volumetric modulated arc therapy (VMAT) was mandatory for all patients. Doses of 70 Gy, 63 Gy, and 56 Gy were delivered to planning target volumes (PTV) at three levels (high, intermediate, and low risk, respectively) in 35 fractions over 7 weeks. An optional RT boost was allowed for patients with residual disease after CCRT. The details regarding RT are provided in the **Supplementary Appendix**. It was recommended that patients commence CCRT within 3 to 4 weeks after the first day of the last cycle of IC.

After completing IC and 16 weeks following RT, tumor responses were assessed with complete physical examination, fiberoptic nasopharyngoscopy, and MRI of the head and neck region, according to the Response Evaluation Criteria in Solid Tumors version 1.1 (RECIST v1.1) (15). Further investigations with contrast-enhanced CT scan of the thorax and abdomen (or PET-CT) were arranged when indicated. Complete physical examination at the end of RT and fiberoptic nasopharyngoscopy with random nasopharyngeal biopsies 8 weeks after the completion of RT were recommended to assess if RT boost was needed. Persistent primary or lymph node disease 16 weeks after the completion of RT was considered a locoregional failure. Acute toxicities during IC and CCRT were evaluated according to the Common Terminology Criteria for Adverse Events version 4.0 (CTCEA v4.0). Late RT-related toxicities were graded according to the Late Radiation Morbidity Scoring Criteria of the Radiation Therapy Oncology Group (16).

In the first 3 years of follow-up, all the patients had regular assessments every 3 months and every 6 months thereafter until death. Whenever possible, locoregional or distant recurrences were confirmed by fine-needle aspiration or biopsy. All endpoints were assessed or confirmed by the primary treating physician.

### End Points

The primary endpoint was progression-free survival (PFS), defined as the time from the start of IC to the first failure at any site (either distant metastasis or locoregional recurrence) or death from any cause, whichever occurred first. Secondary endpoints included overall survival (OS) (the time from the start of IC to death from any cause), locoregional recurrence-free survival (LRFS) (the time from the start of IC to first locoregional failure), distant metastasis-free survival (DMFS) (the time from the start of IC to distant failure), tumor response, compliance to treatment, and severe (grade ≥ 3) acute and late toxicities.

### Statistical Analysis

This non-inferiority trial aimed to evaluate whether the PFS of induction PX-CCRT with reduced cumulative cisplatin dose in LA NPC was not inferior to PFS reported in the NPC-0501 trial. The reported 3-year PFS in the induction PX-CCRT group (Arm 3A) in the NPC-0501 trial was 81% (7). Given the threshold of non-inferior effect δL= -10%, we estimated that 101 NPC cases could achieve 80.1% power by one-side log-rank test at the significance level of 0.05 (17, 18). Assuming 5% early dropout or loss to follow-up, the target accrual was a minimum of 107 patients.

Efficacy analyses were done in both intention-to-treat and per-protocol populations (see the **Supplementary Appendix**). Only patients who received at least 1 cycle of induction PX were included in the safety analyses. Patient demographics, clinicopathologic, and treatment-related factors were reported by descriptive statistics. For each chemotherapy drug of PX, the dose intensity (DI) was calculated as the ratio of the total dose per square meter of the patient, divided by the total treatment duration (mg/m^2^/week). The relative DI was calculated as the ratio of the DI delivered to the DI planned by the protocol. Kaplan–Meier curves were used to describe time-to-event data, and the subgroups were compared with the log-rank tests. All statistical analyses were performed by R software version 3.6.1 and SPSS software version 26.0 (IBM). A two-sided P-value less than 0.05 was considered clinically significant.

## Results

### Patients Characteristics

**Figure 1:**
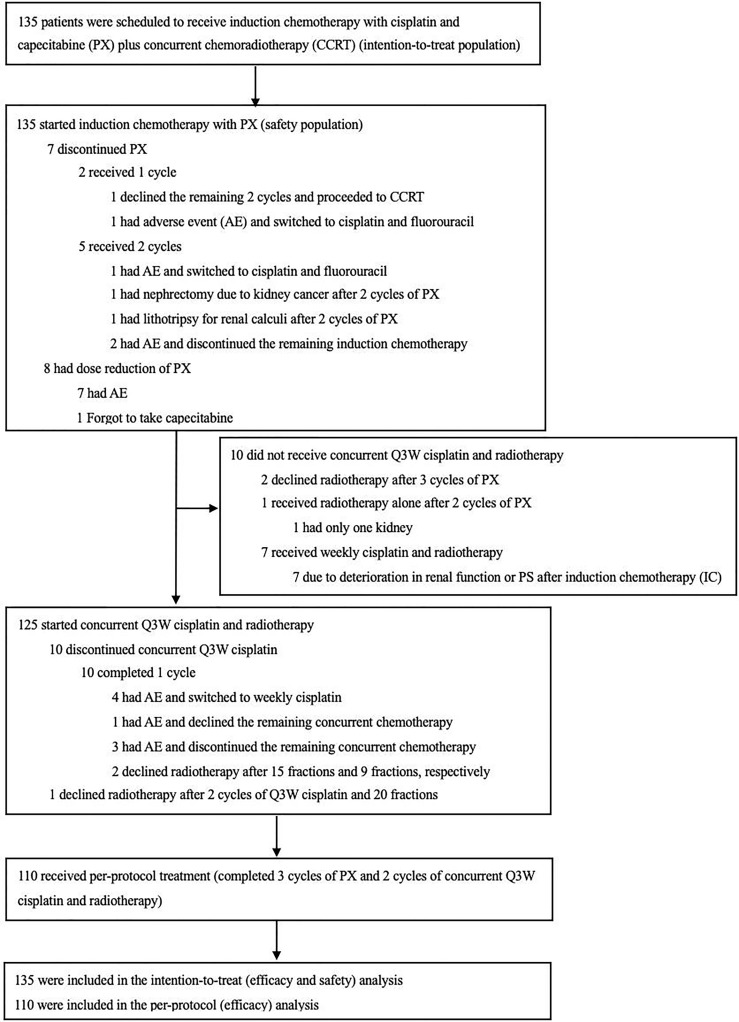
Enrollment and Follow-up.

From January 2015 to September 2019, 135 eligible patients were accrued (**Figure 1**). The median age was 45 years (range 19-70), and 95 (70.4%) patients were male. The detailed characteristics of the patients are shown in **Table 1**.

**Table 1:** Patient clinicopathological characteristics at baseline.

Characteristics	Number of Patients (%)
Total patients	135
Median age (range) – year old	45 (19–70)
Gender	
Male	95 (70.4)
Female	40 (29.6)
Technology	
IMRT	60(44.4)
VMAT	75(55.6)
ECOG performance status	
0	9 (6.7)
1	125 (92.6)
2	1 (0.7)
Tumor category (T)^£^	
T1	11 (8.1)
T2	30 (22.2)
T3	65 (48.1)
T4	29 (21.5)
Lymph node category (N)^£^	
N0	3 (2.2)
N1	16 (11.9)
N2	83 (61.5)
N3	33 (24.4)
Disease stage^£^	
III	78 (57.8)
IVA	57 (42.2)

IMRT, Intensity-modulated radiotherapy; VMAT, Volumetric Modulated Arc Therapy; ECOG, Eastern Cooperative Oncology Group.

^£^Tumor and node categories and disease stage were assessed according to the 8^th^ edition of the American Joint Committee on Cancer–Union for International Cancer Control stage classification system.

### Treatment Tolerance and Compliance

All 135 patients started protocol-defined induction IC (**Figure 1**). A total of 128 patients (94.8%) completed 3 cycles of induction PX. 7 patients (5.2%) failed to complete 3 cycles of induction PX. 2 (1.5%) patients received only one cycle, and 5 (3.7%) patients received two cycles. The reasons for discontinuing PX were shown in **Figure 1**. During IC, 7 patients (5.2%) required dose reductions of cisplatin and/or capecitabine because of neutropenia (n= 1 patient), severe vomiting (n= 2 patients), renal impairment (n= 1 patient), electrolyte disturbance (n= 1 patient), legs edema (n= 1 patient), and common cold (n=1 patient). 1 patient forgot to take the medication. Overall, the median relative DI was 96.2% (interquartile range [IQR], 91.2% to 99.0%) for cisplatin and 93.1% (IQR, 88.7% to 97.2%) for capecitabine (**Table 2**).

**Table 2:** Compliance/tolerance of chemotherapy.

	Induction	Concurrent
No. of cycles of chemotherapy (%)		
3 cycles	128 (94.8)	0 (0)
2 cycles	5 (3.7)	115 (85.2)
1 cycle	2 (1.5)	10 (7.4)
None	0 (0)	3 (2.2)
Cumulative dose (mg/m^2^)		
Cisplatin (Median, IQR)	240 (230-240)	200 (175-200)
Capecitabine (Median, IQR)	5800 (5500-6000)	–

IQR, interquartile range.

Regarding concurrent cisplatin, 125 patients (92.6%) started protocol-defined Q3W cisplatin, 7 patients (5.2%) started weekly cisplatin (at 40mg/m^2^) due to deterioration in renal function or performance status (PS) after IC. Additionally, three patients (2.2%) received no chemotherapy, one patient received RT alone due to a single kidney, and two patients declined RT. A total of 115 of the 135 patients (85.2%) completed 2 cycles of concurrent Q3W cisplatin, and ten patients (7.4%) received only one cycle of concurrent Q3W cisplatin (**Figure 1**). Only one patient (0.7%) switched to concurrent carboplatin due to deterioration in renal function. Overall, 97 of 135 patients (71.9%) received at least 200mg/m^2^ of concurrent cisplatin (including Q3W and weekly cisplatin). 93 patients (68.9%) received the full protocol-defined cumulative cisplatin dose of 440mg/m2 (**Table 2**). However in practice, when we calculate chemotherapy doses based on body surface area, we would round to the nearest whole number. The actual median cumulative dose of cisplatin in the overall patient population was 430mg/m^2^ (IQR, 410 to 440).

Regarding RT, 133 patients (98.5%) started RT, and the remaining 2 patients (1.5%) declined RT after completing 3 cycles of induction PX. A total of 130 patients (96.3%) completed protocol-defined IMRT/VMAT, and another 3 patients (2.2%) declined treatment after 18Gy, 30Gy, and 40 Gy of RT, respectively. On completion of RT, one patient (0.7%) had residual disease of cervical metastatic lymph nodes and received an electron boost to the residual disease. At eight weeks after RT, the pathology-proven residual disease of primary tumor of nasopharynx was detected in one patient (0.7%), and a VMAT boost was delivered. The median time from the start of the last cycle of IC to the commencement of RT was 21 days (IQR, 21 to 24). The median time from the start of the first cycle of IC to the completion of RT was 116 days (IQR, 113 to 121).

### Efficacy

Among the 135 patients recruited to the study, 127 patients (94.1%) achieved a response after IC before the commencement of RT. 15 patients (11.1%) had a complete response (CR), 112 patients (83.0%) had a partial response (PR), and 8 patients (5.9%) had stable disease (SD). No patients had disease progression after IC. At 16 weeks after radiotherapy, all 130 patients (96.3%) who completed the entire course of RT achieved CR. The response of 5 patients (3.8%) who did not complete RT was unavailable (**Table S1 in the Supplementary Appendix**).

At the last follow-up on April 4, 2021, the median follow-up duration was 36.2 months (IQR, 26.1 to 51.8). Twenty-two patients (16.3% of the trial population) experienced disease recurrence, and 8 patients died. Details regarding the patterns of relapse and cause of death are provided in **Table S2 in the Supplementary Appendix**.

**Figure 2:**
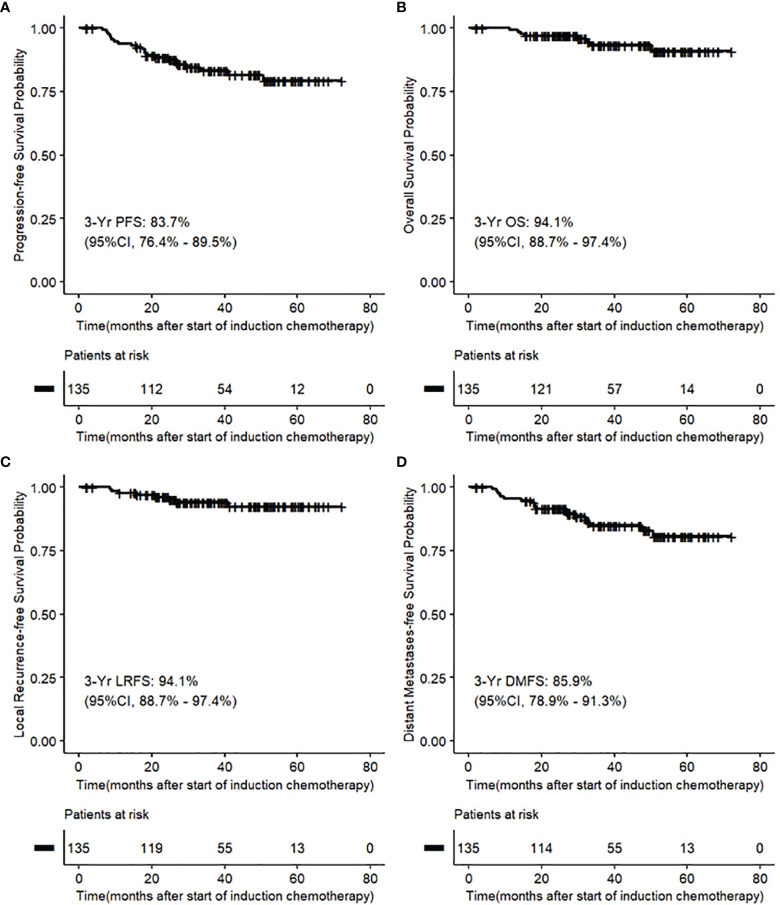
**(A-D)** Kaplan–Meier Analysis of survival outcomes in intention-to-treat population.

For the intention-to-treat population, the 3-year PFS, OS, LRFS, and DMFS were 83.7% (95% confidence interval [CI], 76.4% to 89.5%), 94.1% (95% CI, 88.7% to 97.4%), 94.1% (95% CI, 88.7% to 97.4%), and 85.9% (95% CI, 78.9% to 91.3%), respectively (**Table 3** and **Figures 2A-D**).

**Table 3:** Survival to Treatment.

Variable	Survival
Progression-free survival	
Progression or death — no. (%)	22 (16.3)
Percentage of patients alive and without progression at 3 yr (95% CI)	83.7% (76.4% - 89.5%)
Overall survival	
Death — no. (%)	8 (5.9)
Percentage of patients alive at 3 yr (95% CI)	94.1% (88.7% - 97.4%)
Locoregional recurrence–free survival	
Locoregional recurrence — no. (%)	8 (5.9)
Percentage of patients without locoregional recurrence at 3 yr (95% CI)	94.1% (88.7% - 97.4%)
Distant metastasis–free survival	
Distant metastasis — no. (%)	19 (14.1)
Percentage of patients without distant metastasis at 3 yr (95% CI)	85.9% (78.9% - 91.3%)

CI, confidence interval.

For per-protocol population, the 3-year PFS, OS, LRFS, and DMFS were 85.5% (95% CI, 77.5% to 91.5%), 94.5% (95% CI, 88.5% to 98.0%), 94.5% (95% CI, 88.5% to 98.0%), and 87.3% (95% CI, 79.6% to 92.9%), respectively (**Figures S1A-D in the Supplementary Appendix**).

### Adverse Events

During IC, 29 patients (21.5%) experienced acute grade 3 or 4 (G3-4) adverse events (AEs). Neutropenia was the most common G3-4 AEs (14.8%), followed by electrolyte disturbance (8.9%) and anemia (7.4%). G3-4 capecitabine-related hand-foot syndrome was uncommon (0.7%). During CCRT, 74.1% of patients reported G3-4 AEs. Leukopenia was the most common G3-4 AEs (43.7%), followed by mucositis (28.9%) and anemia (25.9%) (**Table 4**). As for any late toxicity, only 2 out of 135 patients (1.5%) had ≥ G3-4 late RT toxicities (**Table 4**). There was no treatment-related death.

**Table 4:** AEs, according to treatment phase and Grade^#^.

AEs	induction PX	concurrent P +RT	Whole course
	Grade 3-4,	Grade 3-4,	Grade 3-4,
	NO. (%)	NO. (%)	NO. (%)
Any acute AE	29 (21.5)	100 (74.1)	103 (76.3)
Leukopenia	7 (5.2)	59 (43.7)	61 (45.2)
Neutropenia	20 (14.8)	34 (25.2)	45 (33.3)
Neutropenic fever	3 (2.2)	8 (5.9)	11 (8.1)
Infection	1 (0.7)	13 (9.6)	13 (9.6)
Anemia	10 (7.4)	35 (25.9)	37 (27.4)
Thrombocytopenia	3 (2.2)	12 (8.9)	14 (10.4)
Renal function impairment	2 (1.5)	2 (1.5)	4 (3.0)
Electrolyte disturbance	12 (8.9)	14 (10.4)	22 (16.3)
Nausea/vomiting	3 (2.2)	4 (3.0)	6 (4.4)
Diarrhea	2 (1.5)	0 (0.0)	2 (1.5)
Weight loss	0 (0.0)	9 (6.7)	9 (6.7)
Neuropathy	1 (0.7)	3 (2.2)	3 (2.2)
Hand-foot syndrome	1 (0.7)	NA	1 (0.7)
Dermatitis	NA	17 (12.6)	17 (12.6)
Stomatitis (mucositis)	NA	39 (28.9)	39 (28.9)
Any late AE	NA	NA	2 (1.5)
Deafness or otitis	NA	NA	1 (0.7)
Neck tissue damage	NA	NA	1 (0.7)

PX, cisplatin plus capecitabine; CCRT, concurrent chemoradiotherapy; NA, not available.

^#^This analysis was conducted in the safety population, which included patients who began receiving the trial treatment.

### Univariate and Multivariate Cox Regression on PFS

**Figure 3:**
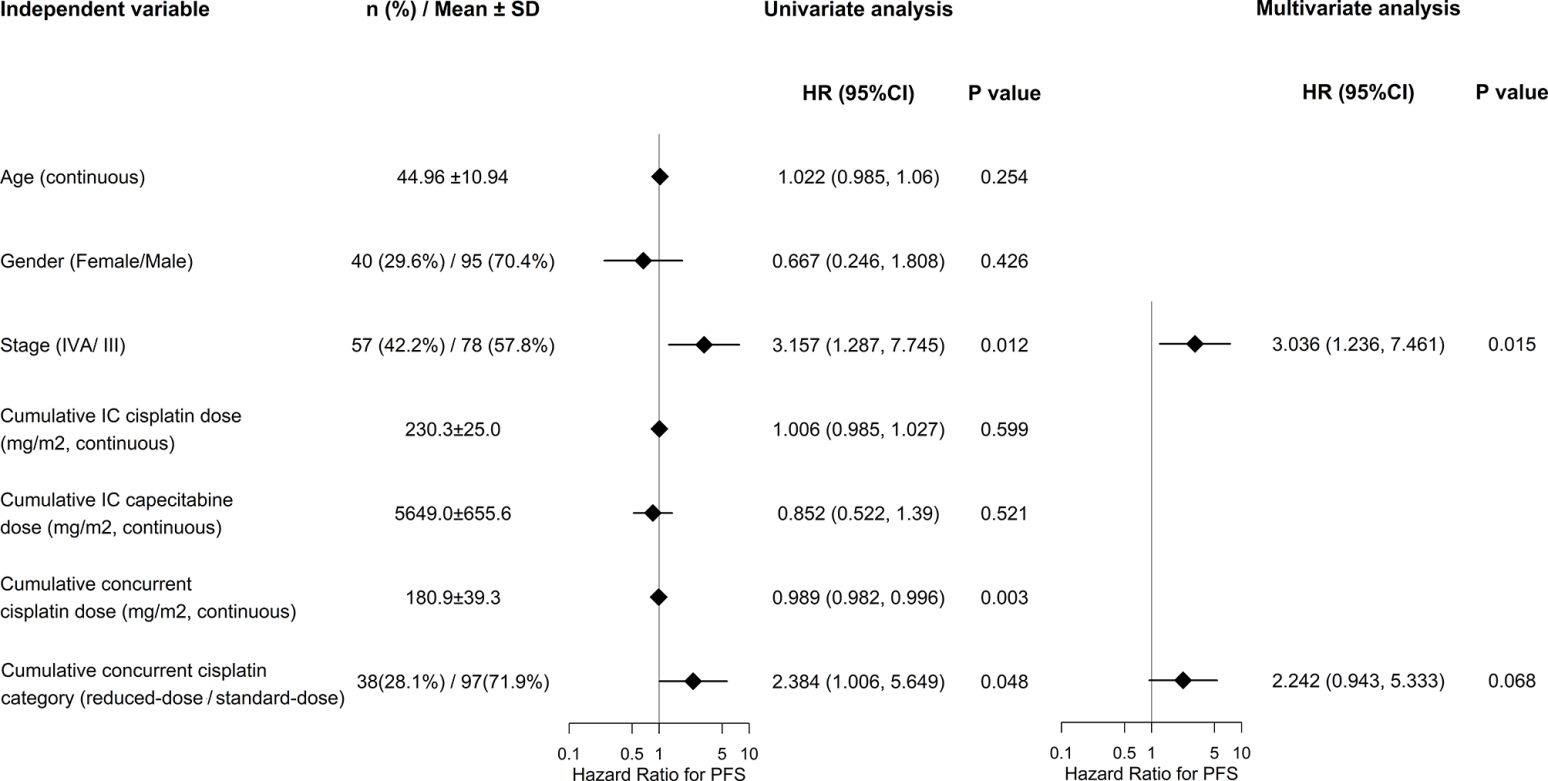
Univariate and multivariate Cox regression on PFS.

With the short follow-up, only univariate and multivariate analyses of PFS rather than OS were performed. As shown in **Figure 3**.

Significant factors of PFS indentified by univariate analyses included tumor stage (IVA/III) and cumulative concurrent cisplatin dose, either as continuous or categorical (reduced-dose/standard-dose) variable (hazard ratio [HR] 3.157, 95% CI 1.287-7.745, P = 0.012; HR 0.989, 95% CI 0.982-0.996, P = 0.003; and HR 2.384, 95% CI 1.006-5.649, P = 0.048; respectively). On multivariate analyses, cumulative concurrent cisplatin dose as categorical variable (HR 2.242, 95% CI 0.943- 5.333, P = 0.068) remained significant when adjusted for tumor stage (HR 3.036, 95% CI 1.236- 7.461, P = 0.015).

## Discussion

The results showed that induction PX-CCRT with a reduced cumulative cisplatin dose in both the induction (80mg/m^2^ x 3 cycles) and concurrent (100mg/m^2^ x 2 cycles) phases was non-inferior to the corresponding induction PX group (Arm 3A) with induction (100mg/m^2^ x 3 cycles) and concurrent (100mg/m^2^ x 3 cycles) cisplatin dose in NPC-0501 trial in terms of PFS (3 yr 83.7% vs. 81%) and OS (3yr 94.1% vs. 91%) in LA NPC, keeping in mind the caveats of cross-study comparisons.

Capecitabine has shown efficacy in IC (8), first-line (19), second-line (20), and maintenance therapy (21) of locoregionally advanced or metastic NPC. IC can minimize the volume of radiation delivered by reducing the tumor size, thus decreasing the radiation dose administered to normal tissue, resulting in improved quality of life (22–24). Theoretically, IC could improve the tolerance to treatment. As expected, the compliance to three cycles of induction PX in our study was numerically higher than in the NPC-0501 trial (94.8% vs. 85%). However, during CCRT, the rate of patients completing 2 cycles of concurrent Q3W cisplatin was numerically lower than in the NPC-0501 trial (85.2% vs. 91%) (7). The most common reason for failing to complete the 2 cycles of Q3W cisplatin was 1) the switch to weekly cisplatin due to deterioration of PS (8.1%), 2) treatment toxicities (3.0%), or 3) withdrawal of consent (3.7%). The proportion of patients receiving at least 200mg/m² of concurrent cisplatin (including Q3W and weekly cisplatin) was 71.9%. Similar to our study, previous studies showed that the cumulative cisplatin dose during CCRT substantially affected locoregional control and OS. Patients who received ≥ 200mg/m² of concurrent cisplatin achieved better OS than those who received a lower dose (13, 14, 25, 26). Although patients received somewhat lower doses of induction and concurrent cisplatin, the survival outcomes of our study were non-inferior to that of NPC-0501. We speculate that this may occur due to the chemotherapy/radiation sensitive nature of NPC (27).

The study published by Mai and colleagues concluded that IMRT plus 2 cycles of concurrent 100 mg/m^2^ cisplatin without induction chemotherapy could be an alternative option for patients with low-risk locoregionally advanced NPC with Epstein-Barr virus (EBV) DNA levels < 4000 copies/ml (28). But for LA NPC, several recently published randomized phase III trials conducted in a similar ethnic patient cohort demonstrated that IC followed by concurrent systemic therapy/RT had better survival benefit than concurrent systemic therapy/RT alone (10–12, 29, 30). Concerning different IC regimens in LA NPC, a network meta-analysis of 9 clinical trials showed that docetaxel + cisplatin (DC), gemcitabine + cisplatin (GP), and PX had favorable OS benefits. GP and PX were the most promising IC regimens to date in the era of IMRT (10). In comparison with induction GP-CCRT, as reported by Zhang and colleagues (11), our trial showed similar results in terms of 3-year survival outcomes and toxicities. The 3-year PFS, OS, LRFS, and DMFS in our study were 83.7%, 94.1%, 94.1%, and 85.9%, respectively; and the corresponding results were 85.3%, 94.6%, 91.8% and 91.1%, respectively. Our locoregional control was better (3-year LRFS: 94.1% vs. 91.8%), and the distant control rate was numerically lower (3-year DMFS: 85.9% vs. 91.1%) than the results in induction GP-CCRT by Zhang et al. (11). This is likely due to fewer patients with T3-4 and more patients with N2-3 in our trial. Compared with the induction GP-CCRT trial by Zhang et al., patients in this study received a lower cumulative dose of cisplatin (430mg/m^2^ vs. 440mg/m^2^), and more patients had N2-3 disease (85.9% vs. 52.9%). Nevertheless, the OS of the two studies were similar (3-year OS 94.1% vs. 94.6%). Concerning toxicities, the incidence of grade 3-4 acute toxicities in the present study was similar to the induction GP-CCRT regimen by Zhang et al. (76.3% vs. 75.7%). The percentage of patients who received protocol-defined cumulative cisplatin dose was 68.9% and 26.4% in the present study and GP-CCRT regimen by Zhang et al., respectively. In general, the reduced cumulative cisplatin treatment schedule in our study produced comparable treatment outcomes compared to other trials and was well tolerated with convenient administration of oral capecitabine. These factors taken together make induction PX-CCRT with reduced cumulative cisplatin dose an appealing treatment option for patients with LA NPC, given the emerging enthusiasm of de-escalation strategy for this disease (31).

Given the paucity of comparative data, the choice of either a gemcitabine-based or capecitabine-based IC regimen could be made based on the expected adverse events matched against the patient’s performance status and comorbidities. The intensity of chemotherapy may be tailored based on various stage subgroups in LA NPC; some studies suggest that patients with stage IV or N2/N3 may benefit from a higher cumulative dose of cisplatin (32, 33).

We have identified some limitations to this study. Firstly, this is a single-arm trial. Prospective randomized controlled clinical trials are needed to confirm the clinical benefit of this reduced cisplatin dose treatment modality. Secondly, we did not include non-anatomical prognostic biomarkers to select eligible participants, especially plasma Epstein-Barr virus (EBV) DNA. Since no prognostic biomarkers have been included in the international staging system for NPC and the treatment recommendation is mainly based on TNM staging. No prognostic biomarkers were included in this study. Thirdly, our trial and the induction-PX regimen in the NPC-0501 trial were not designed random control groups; they were independent and heterogeneous; due to objective reasons, there was no detailed comparison of the patient populations and the results between this study and NPC-0501. Lastly, the median follow-up for the analysis in this study was 3 years, and longer follow-up will be needed to assess long-term survival benefits and late toxic effects fully. Nonetheless, the findings of our study provide valuable data for guiding clinical practice and supporting a reduced cumulative cisplatin dose for future de-escalation clinical trials.

In conclusion, the present study demonstrated that the reduced cumulative cisplatin dose in both induction and concurrent phases could achieve comparable outcomes to the NPC-0501 trial and favorable toxicity profile in LA NPC. However, long-term follow-up and randomized controlled clinical trials are needed to confirm the clinical benefit.

## Data Availability Statement

The original contributions presented in the study are included in the article/**Supplementary Material**. Further inquiries can be directed to the corresponding author.

## Ethics Statement

The studies involving human participants were reviewed and approved by The institutional ethics committee of the University of Hong Kong - Shenzhen Hospital. The patients/participants provided their written informed consent to participate in this study. Written informed consent was obtained from the individual(s) for the publication of any potentially identifiable images or data included in this article.

## Author Contributions

This was an investigator-initiated trial. The first author wrote the first draft of the manuscript, which all the authors reviewed. No pharmaceutical companies were involved in the trial design, data collection or analysis, or manuscript preparation or review. The last author vouches for the completeness and accuracy of the data and the adherence of the trial to the protocol. All authors contributed to the article and approved the submitted version.

## Funding

This project is supported in part by the Health Commission of Guangdong Province, China (NO. B2020100), Shenzhen Science and Technology Program (JCYJ20210324114600002), High Level-Hospital Program, Health Commission of Guangdong Province, China (NO. HKUSZH201902031, HKUSZH201901017, and HKUSZH201901038) and Shenzhen Key Medical Discipline Construction Fund (No. SZXK014).

## Conflict of Interest

The authors declare that the research was conducted in the absence of any commercial or financial relationships that could be construed as a potential conflict of interest.

## Publisher’s Note

All claims expressed in this article are solely those of the authors and do not necessarily represent those of their affiliated organizations, or those of the publisher, the editors and the reviewers. Any product that may be evaluated in this article, or claim that may be made by its manufacturer, is not guaranteed or endorsed by the publisher.
