# Supplementary material for: A Single-Arm Phase 2 Trial on Induction Chemotherapy Followed by Concurrent Chemoradiation in Nasopharyngeal Carcinoma Using a Reduced Cumulative Dose of Cisplatin
Source: Front Oncol. 2022 Apr 27;12:842281. doi: 10.3389/fonc.2022.842281 (PMC9092977; doi:10.3389/fonc.2022.842281)
Supplement: Supplementary file 1 [file DataSheet_1.docx]

**Table of Contents**

The trial’s protocol..................................................................................................2 - 13

Details of the induction chemotherapy dose modifications.........................................14

Details of the concurrent chemotherapy dose modifications.......................................15

Specifics of Radiotherapy treatment.....................................................................16 - 22

Table S1 Response to treatment...................................................................................23

Table S2 Disease recurrence distribution and cause of death......................................24

Figure S1A-S1D Survival to Treatment of per-protocol population............................25


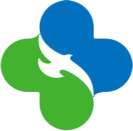
 The University of Hong Kong-Shenzhen Hospital

**The trial’s protocol**

| **A single-arm phase 2 trial on induction chemotherapy followed by concurrent chemoradiation in****nasopharyngeal carcinoma using** **a reduced cumulative dose of cisplatin** |
| --- |

## Objective

| 1. | Objective | | |
| --- | --- | --- | --- |
|  | 1.1 | To provide protocol on the trial |  |

## Index

2. Index

3 Diagnosis & workup

4 Staging

1. Management
   1. Primary treatment strategy for different stages
   2. Radiotherapy
   3. Assessment of response to primary treatment
   4. Treatment of residual tumour at first post–RT assessment and persistent disease at final post–RT assessment
   5. Follow-up
   6. Management of loco-regional recurrence
   7. Management of metastatic disease

**Inclusion criteria:**

- Age 18 to 75 years;
- Previously untreated, newly histologically confirmed non-keratinizing NPC;
- Stage III to stage IVB disease using the 7th edition of the American Joint Committee on Cancer–Union for International Cancer Control (AJCC-UICC TNM-7) before 2018 or stage III-IVA disease based on AJCC-UICC TNM-8 since 2018 (except T3N0).
- An Eastern Cooperative Oncology Group (ECOG) performance status (PS) ≤ 2;
- Adequate hematologic, hepatic, and renal function.

**Exclusion criteria:**

- Treatment with palliative intent;
- A history of prior malignancy;
- A history of previous chemotherapy, radiotherapy, or surgery (except diagnostic procedures) to the primary tumour or nodes;
- Pregnancy or lactation;
- Any severe comorbidity.

## Diagnosis and workup

| 3. | **Diagnosis and workup** | |
| --- | --- | --- |
|  | | |
|  | 3.1 | **Diagnosis and histological classification** |
|  |  | - History including family history, smoking history and assessment of co-morbidity Biopsy for histological confirmation |
|  |  | - WHO classification system |
|  | | |
|  | 3.2 | **Primary Investigations** |
|  |  | - CBP, RFT, LFT, LDH, Plasma EBV DNA |
|  |  | - T4/TSH, morning cortisol |
|  |  | - Endoscopic examination |
|  |  | - MRI of NP and neck |
|  |  | - PET-CT recommended (may consider other metastatic work-up if stage I) |
|  | **3.3** | **Pre-treatment preparation** |
|  |  | - Dental check |
|  |  | - Audiometric assessment |
|  |  | - Nutritional assessment |

## Staging system

| 4. | **Staging system** | |
| --- | --- | --- |
|  | | |
|  |  | AJCC/UICC 8^th^ edition of TNM Staging Classification (TNM-8) |

## Management

| 5. | **Management** | |
| --- | --- | --- |
|  | | |
|  | 5.1 | **Primary treatment strategy** |
|  |  | **Stage III-IVA** |
|  |  | *High risk:* (except T3N0 and EBV DNA < 500 copies/mL)  Radical RT plus induction-concurrent chemotherapy  Induction chemotherapy  PX q3wk for 3 cycles  Cisplatin 80mg/m2 IV D1 (Change to carboplatin AUC x 5 if CrCl 30-60)  Capecitabine (Xeloda) 1000 mg/m2 twice daily (i.e. 2000mg/m2/day) D1- D14 (i.e. total 14 days)  Concurrent chemotherapy  concurrent Cisplatin q3wk x 2 cycles  Cisplatin 100 mg/m2 iv infusion D1  ( start or switch to weekly cisplatin at 40mg/m^2^ if deterioration in renal function or PS) |
|  |  |  |
|  | 5.2 | **Radiotherapy**  Repeat Investigations prior to RT Planning if induction chemotherapy given   - CBP, RFT, LFT, LDH, Plasma EBV DNA - MRI of NP and neck   Treatment delivery |
|  |  | - Beam: 6 MV photon - Technique: IMRT / VMAT / tomotherapy with image guidance - Adaptive RT if affordable by the patient |
|  |  |  |
|  |  | Target delineation & OAR dose constraint:   - Follow the International Consensus Guidelines^[2, 3]^ (see Appendix) - Tumor targets after induction chemotherapy should take into consideration the tumor extent both at diagnosis and post-induction chemotherapy |
|  |  | Dose Prescription: |
|  |  | - 35 fractions, 5 fr/wk |
|  |  | Total dose and dose / fraction: |
|  |  | - PTV_70: 70 Gy (100% IL) at 2 Gy/fr |
|  |  | - PTV_63: 63 Gy at 1.8 Gy/fr |
|  |  | - PTV_56: 56 Gy at 1.6 Gy/fr |
|  |  | Adaptive RT  Mid-course assessment with MRI and planning CT after 36 Gy  Re-planning if substantial changes in   - GTVp and GTVn – volume & dose - Dose to brainstem, spinal cord and optic chiasm - Body contour – fitness of immobilization cast   Boost for residual neck node at last fraction |
|  |  | - Beam: 9 MeV electron - Involved field - 1cm around residual node - Dose: 2 Gy at 90% IL, 5 fr/wk, for 5 fractions |
|  |  |  |
|  | **5.3** | **Assessment of response to primary treatment** |
|  |  | ***First post-RT assessment*** |
|  |  | Time: 8 weeks after completion of primary RT |
|  |  | Investigations: |
|  |  | - Physical examination for residual lymph nodes |
|  |  | - Endoscopic examination & biopsy |
|  |  | - Plasma EBV DNA |
|  |  |  |
|  |  | Final post-RT assessment |
|  |  | Time: 16 weeks after completion of primary RT |
|  |  | Investigations: |
|  |  | - MRI NP and neck |
|  |  | - Plasma EBV DNA |
|  |  | - Progress PET recommended especially if EBV-DNA >0 |
|  |  |  |
|  | **5.4** | **Treatment of residual tumor at first post–RT assessment** |
|  |  | A) Local residual |
|  |  | RT boosting |
|  |  | - PTV for 100% IL: gross residual tumor with 2 mm margin |
|  |  | - Dose: 2 Gy/Fr at 100%, 5 Fr/week, for 10 Fr (i.e. boost dose = 20 Gy) |
|  |  | - Attempt to achieve 95% dose to residual GTV as far as possible (absolute dose constraint to brainstem, spinal cord and optic chiasm: ≤ 15% IL) |
|  |  | B) Nodal residual |
|  |  | - Refer ENT for assessment of neck dissection |
|  |  |  |
|  |  | **Treatment of persistent local tumor at final assessment** |
|  |  | Individualized – |
|  |  | If operable: consider surgery |
|  |  | If inoperable: consider chemotherapy if medically fit and keen |
|  |  | **Management of patients with persistent detectable EBV-DNA**  Consider metronomic chemotherapy if medically fit and keen^[4]^ |
|  | **5.5** | **Follow up** |
|  |  | \| 1^st^- 2^nd^ years \| every 2-3 months \| \| --- \| --- \| \| 3^rd^ – 4^th^ year \| every 3-4 months \| \| 5^th^ year \| every 4-6 months \| \| 6^th^ – 10^th^ year \| every 6 months \| \| after 10 years \| Every 1 year \| |
|  |  | Assessment   - Physical and endoscopic exam - Plasma EBV DNA - Hormonal profile - T4/TSH, morning cortisol (at least yearly) - MRI of NP and neck - every 6 months for first 5 years, then yearly for 5-10 years - PET-CT - yearly for first 3 years - More frequent assessments if suspicious symptom or detectable EBV-DNA   Survivorship and rehabilitation   - Annual dental check - ENT follow up and assessment - Referral to speech therapist if dysphagia/regurgitation problem |
|  | **5.6** | **Management of loco-regional recurrence** |
|  |  | Individualized **-** Radical as far as possible |
|  |  | Surgery (preferred if operable): |
|  |  | - Local failure: nasopharyngectomy |
|  |  | - Nodal failure: ipsilateral neck dissection |
|  |  | - Add post-op. RT if resection margin <2 mm |
|  |  |  |
|  |  | Inoperable: |
|  |  | *Radical* |
|  |  | Induction chemotherapy for 3-4 cycles, then reassess for radical re-irradiation (+/- concurrent chemotherapy) if: |
|  |  | - No pre-existing G3-4 RT related complication (except hearing loss and xerostomia) - Tumor volume ≤40 cc at recurrence - No disease progression after induction chemotherapy - Preferably with adequate sparing of critical neurological structures |
|  |  |  |
|  |  | *Palliation* |
|  |  | Palliative chemotherapy for 6 cycles |
|  |  | Consider local RT if symptomatic or bulky disease |
|  |  |  |

|  |  | Re-irradiation |
| --- | --- | --- |
|  |  | - Technique: IMRT / VMAT / tomotherapy with image guidance - Target delineation: see Appendix II - Dose prescription: |
|  |  | \|  \| Dose / fraction at PTV \| Total dose \| \| --- \| --- \| --- \| \| *Radical re-irradiation* \| \| \| \| No surgery / gross residual after surgery \| 1.2 Gy/fr, BID (> 6 hr interfraction interval), 10 fr/week \| 64.8 Gy in 54 Fr  (Eq. total dose – 60.5 Gy) \| \| Microscopic residual / close margin (< 2 mm) after surgery \| 1.2 Gy/fr, BID (> 6 hr interfraction interval), 10 fr/week \| 57.6 Gy in 48 Fr  (Eq. total dose – 53.8 Gy) \| \| *Palliative re-irradiation* \| \| \| \|  \| 2 Gy/ Fr, 5 Fr/ wk \| 50 Gy in 25 Fr \| |
|  |  | - Dose acceptance criteria (see appendix): |
|  |  | - - Cumulative lifetime BED dose ≤130% (desirable) to 150% (acceptable) of maximum tolerance dose by 1 course   - Attempt to achieve 95% dose to recurrent GTV as far as possible (absolute dose constraint confined to brainstem, spinal cord and optic chiasm) |
|  |  |  |
|  | **5.7** | **Treatment of distant metastasis**  Gauge prognosis:   - Host: age, gender, PS, co-morbidities - Metastases:   - Metastatic sites: lung, bone, liver, distant LN, others   - Total number of metastatic lesions - Time from initial diagnosis - Blood tests:   - CBP (Hb, Neutrophil, Lymphocyte, Monocyte),   - LDH,   - LFT (including GGT), ALP   - Plasma EBV DNA |
|  |  | Management: |
|  |  | 1. Oligo-metastases [≤ 5 metastatic lesions (preferably ≤ 3), no. of involved organs ≤ 2, and size of any metastatic lesion preferably ≤ 5 cm]: |
|  |  | 1. Chemotherapy for 6 cycles + ablative treatment to metastatic sites (surgery, RFA or SBRT) |
|  |  | 1. Best supportive care if poor PS / significant co-morbidity 2. Extensive metastases    1. Palliative chemotherapy – choice individualized based on prior treatment and response (see Appendix)    2. Best supportive care if poor PS / significant co-morbidity |
|  |  |  |

**Details of the induction chemotherapy dose modifications (PX)**

**Scheme:**

PX q3wk for 3 cycles

Cisplatin 80mg/m2 IV D1

Capecitabine (Xeloda) 1000 mg/m2 twice daily (i.e. 2000mg/m2/day) D1- D14 (i.e. total 14 days)

Change to carboplatin area under the curve (AUC) x 5 if creatinine clearance (CrCl) 30-60

Withhold chemotherapy until absolute neutrophil count (ANC) ≧1.5 and platelet ≧100 and gastrointestinal (GI) toxicities ≦ grade 1

**Dose modifications:**

75% of the original dose of chemo if ≧ grade 3 toxicities (except nausea/vomiting) or ≧ 2 weeks delay in chemotherapy dosing due to any toxicities

75 % of the original dose of Xeloda alone if ≧ grade 2 hand-foot syndrome (HFS)

**Details of the concurrent chemotherapy dose modifications (Cisplatin)**

**Scheme:**

Chemo-RT Scheme: concurrent Cisplatin q3wk x 2 cycles

Cisplatin 100 mg/m2 iv infusion D1

(start or switch to weekly cisplatin at 40mg/m^2^ if there is evidence of deterioration in renal function or PS)

Start new cycle if ANC >or= 1.5 and plt >or= 100

**Dose modification:**

Based on complete blood count nadir

ANC 0.5-1 and/or platelet 50 - 74, give 75% of the original dose

ANC <0.5 and/or platelet <50, give 50% of the original dose

If CrCl is between 50-59ml/min, given 75% of the original dose

If CrCl <50ml/min, omit CDDP

**Specifics of Radiotherapy treatment**

| Repeat Investigations prior to radiotherapy (RT) Planning if induction chemotherapy given   - CBP, RFT, LFT, LDH, Plasma EBV DNA - MRI of NP and neck   Treatment delivery |
| --- |
| - Beam: 6 MV photon - Technique: IMRT / VMAT with image guidance |
|  |
| Target delineation & OAR dose constraint:   - Follow the International Consensus Guidelines ^1,2^(see Reference) - Tumor targets after induction chemotherapy should be taken into consideration - The tumor extent both at diagnosis and post-induction chemotherapy |
| Dose Prescription: |
| - 35 fractions, 5 fr/wk |
| Total dose and dose / fraction: |
| - PTV_70: 70 Gy (100% IL) at 2 Gy/fr |
| - PTV_63: 63 Gy at 1.8 Gy/fr |
| - PTV_56: 56 Gy at 1.6 Gy/fr |
| Boost for residual neck node at last fraction |
| - Beam: 9 MeV electron - Involved field - 1cm around residual node - Dose: 2 Gy at 90% IL, 5 fr/wk, for 5 fractions |
|  |
| **Assessment of response to primary treatment** |
| ***First post-RT assessment*** |
| Time: 8 weeks after completion of primary RT |
| Investigations: |
| - Physical examination for residual lymph nodes |
| - Endoscopic examination & biopsy |
| - Plasma EBV DNA |
|  |
| Final post-RT assessment |
| Time: 16 weeks after completion of primary RT |
| Investigations: |
| - MRI NP and neck |
| - Plasma EBV DNA |
| - Progress PET recommended especially if EBV-DNA >0 |
|  |
| **Treatment of residual tumor at first post–RT assessment** |
| A) Local residual |
| RT boosting |
| - PTV for 100% IL: gross residual tumor with 2 mm margin |
| - Dose: 2 Gy/Fr at 100%, 5 Fr/week, for 10 Fr (i.e. boost dose = 20 Gy) |
| - Attempt to achieve 95% dose to residual GTV as far as possible (absolute dose constraint to brainstem, spinal cord and optic chiasm: ≤ 15% IL) |
| B) Nodal residual |
| - Refer to ENT for assessment of neck dissection |
|  |
| **Treatment of persistent local tumor at final assessment** |
| Individualized – |
| If operable: consider surgery |
| If inoperable: consider chemotherapy if medically fit and keen |
|  |

Radiotherapy technology

Immobilization

- Thermoplastic head and shoulder mask, with headrest in baseboard embedded on Vaclock shoulder support
- Supine
- No mouth-bite
- Neck slightly extended (lower border of chin above the cranial border of hyoid bone)

CT simulation and image fusion

Planning CT with IV contrast, 2 mm slice thickness from skull vertex to 2 cm below clavicles

Image registration and fusion with MRI +/- PET-CT if available

Register both scans at diagnosis and post-induction chemotherapy (if given)

Target delineation

A. Gross Tumor Volume (GTV)

- GTVp = primary tumor
- GTVn = nodal disease
- Post-induction:
- Register both MRI scans at diagnosis and post-induction chemotherapy
- Anatomical structures and OAR should be based on post-chemotherapy status
- If feasible to achieve total therapeutic dose without exceeding tolerance dose to critical OAR: Use pre-induction GTV for contouring CTV_70 (Ref. Salama et al.^3^)
- If compromise is needed for tumor infiltrating/abutting neurological structures: Use post-induction GTV for contouring CTV_70, but ensure complete coverage of pre-induction GTV to ≥64 Gy (Ref. Yang et al.^4^)

B. Clinical Target Volume (CTV)

*CTV_70*

- CTVp_70 = GTVp + 5 mm^a^ + anatomical editing to include whole NP^b^
- Minimal margin^a^ – GTVp + 1 mm if GTVp abutting critical neurological OAR
- Landmark for NP^b^:
- Cranial border - base of skull
- Anterior border - junction with nasal choana superiorly and medial pterygoid plate inferiorly
- Lateral border - medial border of parapharyngeal space
- Caudal border - caudal border of C1 vertebra, and spare soft palate if not involved
- CTVn_70 = GTVn + 5 mm

*CTV_63 (high-risk subclinical sites)*

- CTVp_63 = GTVp + 10 mm^a^ + anatomical editing to include high-risk subclinical disease
  - Minimal margin^a^ – GTVp + 2 mm if GTVp abutting critical neurological OAR
- High-risk subclinical disease^b^:
- Nasal cavity: posterior fourth (≥5 mm from choana)
- Maxillary sinuses: posterior fourth (≥5 mm from post. wall)
- Posterior ethmoid sinus: include vomer
- Parapharyngeal spaces: entire spaces to the lateral border of styloid processes
- Pterygoid fossae & pterygopalatine fossae
- Sphenoid sinus: inferior part if T1-2; entire sinus if T3-4
- Clivus: anterior third if no gross clivus invasion; the whole clivus if T3-4
- Base of skull: cover foramina ovale, rotundum, lacerum & petrous tips
- Cavernous sinus: if T3-4 (involved side only)
- CTVn_63 = GTVn + 10 mm (15 mm if bulky LN or extracapsular extension; 5 mm if dubious LN with shortest axis <1 cm) + elective nodal regions
- Elective nodal regions:

| All cases | Bilateral retropharyngeal |
| --- | --- |
| Node +ve / suspicious | level II + III + Va  Ipsilateral one level below involved level |
| Ib or SMG involvement / structures that drain to level Ib^a^ / level II LNs with ECE or axial diameter >2 cm / violation of the neck before RT | Ipsilateral level Ib |

^a^oral cavity, anterior half of nasal cavity or maxillary sinus

*CTV_56 (low risk subclinical sites)*

| Node -ve (confirmed by PET) | Bilateral level II + III + Va |
| --- | --- |
| Node +ve / suspicious | Ipsilateral level IV + Vb |

C. Planning Target Volume (PTV)

- Cranial to lower border of C1 vertebra: + 3 mm margin
- Caudal to lower border of C1 vertebra: + 5 mm margin
- Spare ≥3 mm dermal tissues (5 mm from cast) if no skin involvement

D. Planning Organ at Risk Volume (PRV)

- Spinal cord – 2 mm margin (5 mm if do not affect tumor doses)
- Brainstem, optic chiasm & nerve – 2 mm margin (3 mm if do not affect tumor dose)

Normalization

- 100% of PTV_70 is covered by the ≥95% isodose level (Ideal)
- ≥95% of PTV_70 is covered by the 100% isodose level (Acceptable)

Dose specification for tumor targets and OAR^5^

|  | Desirable criteria | Acceptable criteria |
| --- | --- | --- |
| **Priority 1: Critical OAR** | | |
| Brainstem (PRV) D0.03cc | ≤54 Gy | ≤60 Gy |
| Spinal cord (PRV) D0.03cc | ≤45 Gy | ≤50 Gy |
| Optic chiasm (PRV) D0.03cc | ≤54 Gy | ≤60 Gy |
| **Priority 2: Tumor targets and other important neurological OAR** | | |
| GTV-P & GTV-N | Min ≥68.6 Gy (98% dose) | Min ≥66.5 Gy (95% dose) |
| PTVmin | ≥95% PTV 100% or  ≥99% PTV ≥93% dose | 95% PTV ≥95% dose |
| PTVhotspot | <5%PTV70 ≥75 Gy or  <10%PTV70 ≥77 Gy | <10%PTV70 ≥75 Gy or  <20%PTV70 ≥77 Gy |
| Temporal lobe (PRV) D0.03cc | <65 Gy for T1/2  <70 Gy for T3/4 | ≤72 Gy |
| **Priority 3: Intermediate-risk OAR** | | |
| Optic nerve* (PRV) D0.03cc | ≤54 Gy | ≤60 Gy |
| Brachial Plexus D0.03cc | <66 Gy | ≤70 Gy |
| Lens (PRV) D0.03cc | ≤6 Gy | ≤15 Gy |
| Eyeball | Mean ≤35 Gy | ≤50 Gy (D0.03cm^3^) |
| **Priority 4: Low-risk OAR** | | |
| Parotid gland (mean) | <26 Gy | <30 Gy (at least 1 gland) |
| Pituitary D0.03cc | ≤60 Gy | ≤65 Gy |
| Mandible & TM joint D2% | ≤70 Gy | ≤75 Gy |
| Cochlea (mean) | ≤45 Gy | ≤55 Gy |
| Glottic larynx (mean) | ≤35 Gy | ≤50 Gy (D2%) |
| Postcrioid pharynx, esophagus (within field) (mean) | ≤45 Gy | ≤55 Gy |
| Oral Cavity (excluding PTV) (mean) | <40 Gy | <50 Gy |
| Submandibular gland (mean) | <35 Gy |  |
| Thyroid | V_50_<60% | VS_60_ <10 cc |

* if bilateral optic nerve

Reference

1. Lee AW, Ng WT, Pan JJ, et al. International guideline for the delineation of the clinical target volumes (CTV) for nasopharyngeal carcinoma. Radiother Oncol 2018;126:25-36.

2. Lee AW, Ng WT, Pan JJ, et al. International Guideline on Dose Prioritization and Acceptance Criteria in Radiation Therapy Planning for Nasopharyngeal Carcinoma. Int J Radiat Oncol Biol Phys 2019;105:567-80.

3. Salama JK, Haddad RI, Kies MS, et al. Clinical practice guidance for radiotherapy planning after induction chemotherapy in locoregionally advanced head-and-neck cancer. Int J Radiat Oncol Biol Phys 2009;75:725-33.

4. Yang H, Chen X, Lin S, et al. Treatment outcomes after reduction of the target volume of intensity-modulated radiotherapy following induction chemotherapy in patients with locoregionally advanced nasopharyngeal carcinoma: A prospective, multi-center, randomized clinical trial. Radiother Oncol 2018;126:37-42.

5. Lee AW, Ng WT, Pan JJ, et al. International Guideline on Dose Prioritization and Acceptance Criteria in Radiation Therapy Planning for Nasopharyngeal Carcinoma. Int J Radiat Oncol Biol Phys 2019;105:567-80.

**Table S1 Response to treatment^§^**

| Response | Response to induction chemotherapy — no. (%)^§^ | Response to whole treatment — no. (%) |
| --- | --- | --- |
| CR | 15 (11.1) | 130 (96.3) |
| PR | 112 (83.0) | 0 (0.0) |
| SD | 8 (5.9) | 0 (0.0) |
| PD | 0 (0.0) | 0 (0.0) |
| NA | 0 (0.0) | 5 (3.7)^¤^ |
| ORR (CR + PR) | 127 (94.1) | 130 (96.3) |

CR, complete response, PR, partial response, SD, stable disease, PD, progression disease, NA, not available, ORR, overall response rate

^¤^ These patients were those who did not finish the per-protocol radiotherapy.

Among 135 patients, 127 patients (94.1%) achieved a response after IC before the commencement of RT; 15 patients (11.1%) had a complete response (CR), 112 patients (83.0%) had a partial response (PR), and 8 patients (5.9%) had stable disease (SD). No patient had disease progression after IC. At 16 weeks after radiotherapy, all 130 patients (96.3%) who completed the entire course of RT had a CR. The response of other 5 patients (3.8%) who did not complete RT was not available (Table S1 in the Supplementary Appendix).

**Table S2 Disease recurrence distribution and cause of death**

| Disease recurrence | No. (%) |
| --- | --- |
| Distant |  |
| Bone | 5 (3.7) |
| Lung | 1 (0.7) |
| Liver | 3 (2.2) |
| Other | 0 (0.0) |
| Multiple sites | 5 (3.7) |
| Locoregional |  |
| Local alone | 0 (0.0) |
| Regional alone | 0 (0.0) |
| Local + regional | 3 (2.2) |
| Distant + Locoregional |  |
| Distant + local | 1 (0.7) |
| Distant + regional | 2 (1.5) |
| Distant + local + regional | 2 (1.5) |
| Death |  |
| Cancer-specific | 8 (5.9%) |
| Non-cancer-specific | 0 (0.0%) |

At the last follow-up on April 4, 2021, the median follow-up duration was 36.2 months (IQR, 26.1 to 51.8). A total of 22 events (16.3% of the trial population) of recurrence or death were recorded and 8 of them had died. Details regarding the patterns of relapse and cause of death are provided in Table S2 in the Supplementary Appendix.

**Figure S1A-S1D Survival to Treatment of per-protocol population**


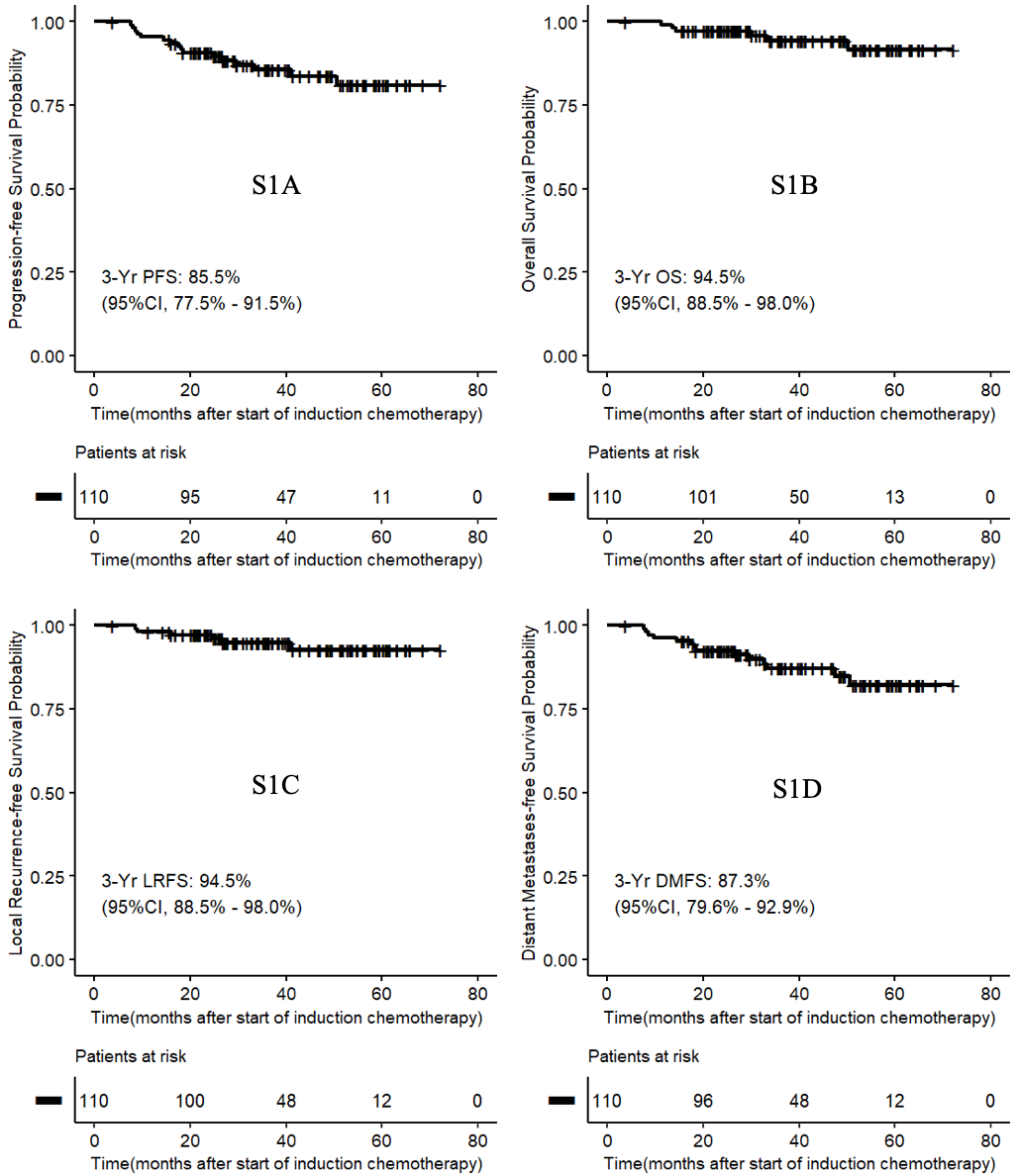


For per-protocol trial population, Kaplan–Meier Analysis showed the 3-year PFS, OS, LRFS, and DMFS were 85.5% (95% CI, 77.5% to 91.5%), 94.5% (95% CI, 88.5% to 98.0%), 94.5% (95% CI, 88.5% to 98.0%), and 87.3% (95% CI, 79.6% to 92.9%), respectively.
